# 

**Published:** 1905-03

**Authors:** 


### BOOKS RECEIVED.

The Influence of Growth on Congenital and Acquired Deformities. By ADONIRAM BROWN JUDSON, M.D., Orthopedic Surgeon to the Out-Patient Department, New York Hospital, 1878-1903. Octavo, pp. 286. Illustrated. New York: William Wood & Company. 1905.

Transactions of the Medical Association of the State of Alabama. Annual meeting held at Mobile, April 19-22, 1904. LEWIS C. MORRIS, M.D., Secretary.

International Clinics. A Quarterly of Illustrated Clinical Lectures and especially prepared articles on Treatment, Medicine, Surgery, Neurology, Pediatrics, Obstetrics, Gynecology, Orthopedics, Pathology, Dermatology, Ophthalmology, Otology, Rhinology, Laryngology, Hygiene and other topics of interest to students and practitioners. By leading members of the medical profession throughout the world. Edited by A. O. J. KELLY, A.M., M.D., Philadelphia. Volume IV. Fourteenth series. 1905. Philadelphia: J. B. Lippincott Company. (Cloth, $2.00.)

Studies in the Psychology of Sex—Sexual Selection in Man. I. Touch. II. Smell. III. Hearing. IV. Vision. By HAVELOCK ELLIS. Pages xii. 270. Sold only by subscription to physicians, lawyers, and scientists. Philadelphia: F. A. Davis Company. 1905. (Cloth, $2.00 net.)

Practical Pediatrics. A Manual of the Medical and Surgical Diseases of Infancy and Childhood. By Dr. E. GRAETZER, Editor of the *Centralblatt Fur Kinderheilkunde.* Authorised translation, with numerous additions and notes. By Herman B. Sheffield, M.D., Instructor in Diseases of Children, and Attending Pediatrist, New York Post-Graduate Medical School and Hospital. Pages xii. 544. Crown octavo. Philadelphia: F. A. Davis Company. 1905. (Flexible cloth, $3.00.)

Eye, Ear, Nose and Throat Nursing. By A. EDWARD DAVIS, A.M., M.D., Professor of Diseases of the Eye in the New York Post-Graduate Medical School and Hospital, and Beaman Douglass, M.D., Professor of Diseases of the Nose and Throat in the New York Post-Graduate Medical School and Hospital. With 32 illustrations. Pages xvi. 318. Philadelphia: F. A. Davis Company. 1905. (Price, $1.25.)

Saunders's Medical Hand-Atlases. Atlas and Epitome of Operative Ophthalmology. By Dr. O. HAAB, of Zürich. Edited, with additions, by George E. de Schweinitz, M.D., Professor of Ophthalmology in the University of Pennsylvania. With 30 colored lithographic plates, 154 text-cuts, and 377 pages of text. Philadelphia, New York, London: W. B. Saunders & Company. 1905. (Cloth, $3.50 net.)

A Textbook of Legal Medicine. By FRANK WINTHROP DRAPER, A.M., M.D., Professor of Legal Medicine in Harvard University. Octavo, 573 pages, fully illustrated. Philadelphia, New York, London: W. B. Saunders & Company. 1905. (Cloth, $4.00 net.)

Bacteriology and Surgical Technic for Nurses. By EMILY M. A. STONEY, Superintendent of the Training School for Nurses, Saint Anthony's Hospital, Rock Island, Ill. Second edition, thoroughly revised and much enlarged by Frederic R. Griffith, M.D., Surgeon, Fellow of the New York Academy of Medicine. Duodecimo volume of 278 pages, fully illustrated. Philadelphia, New York, London: W. B. Saunders & Company. 1905. (Cloth, $1.50 net.)

The Modern Mastoid Operation. By FREDERICK WHITING, A.M., M.D., Professor of Otology, Cornell University Medical College. Royal square octavo, pp. xiii. 353. Illustrated by 25 half-tone and 23 key plates made from original drawings. Philadelphia: P. Blakiston's Son & Company. 1905. (Half morocco, $6.00 net.)

